# Low muscle mass affect hip fracture treatment outcomes in older individuals: a single-institution case-control study

**DOI:** 10.1186/s12891-021-04143-6

**Published:** 2021-03-09

**Authors:** Hiroki Iida, Taisuke Seki, Yoshihito Sakai, Tsuyoshi Watanabe, Norimitsu Wakao, Hiroki Matsui, Shiro Imagama

**Affiliations:** 1 Department of Orthopedic Surgery, Nagoya University Graduate School of Medicine, Nagoya, Aichi Japan; 2 Department of Orthopedic Surgery, National Center for Geriatrics and Gerontology, Obu, Aichi Japan

**Keywords:** Hip fracture, Case–control studies, Sarcopenia

## Abstract

**Background:**

Although sarcopenia has been known as a risk factor for hip fracture, only a few reports have described the impact of muscle mass on hip fracture treatment outcomes. The current study aimed to investigate the impact of muscle mass on hip fracture treatment outcomes.

**Methods:**

This case–control study involved 337 patients (67 males and 270 females) with hip fracture aged ≥65 years (mean age: 84.1 ± 7.1 years) who underwent orthopedic surgery from January 2013 to June 2019. The mean follow-up period was 17.1 (1–60) months. Upon admission, all patients were assessed for low muscle mass according to the Asian Working Group for Sarcopenia criteria (male, SMI < 7.00 kg/m^2^; female, SMI < 5.40 kg/m^2^) using dual-energy X-ray absorptiometry. Treatment outcomes (stays at acute care institutions, hospital mortality, the Barthel index at discharge, and home discharge rates, and one-year mortality) were compared between patients with and without low muscle mass by Student’s t-test, Mann-Whitney U test and the Pearson Chi-Square test. A multivariate logistic regression model was used to calculate adjusted odds ratios (ORs) with 95% confidence intervals (CIs) for factors related to low muscle mass. Kaplan–Meier survival curves on one-year mortality of hip fracture patients for those with and without low muscle mass were prepared, and log-rank tests were performed. Furthermore, we determined whether low muscle mass was a risk factor for one-year mortality in hip fracture patients using a Cox proportional hazards model.

**Results:**

The prevalence of low muscle mass in patients with hip fracture was 231(68.5%). Those with low muscle mass had a lower Barthel index (*P* < 0.0001), hospital discharge rate (*P* = 0.035) and higher one-year mortality (*P* = 0.010). Cox proportional hazards regression analysis adjusted for age and sex found that low muscle mass was a risk factor for one-year mortality (hazard ratio, 3.182, 95% confidence interval, 1.097–9.226, *P* = 0.033).

**Conclusions:**

Patients with hip fracture who had low muscle mass had a lower Barthel index, lower home discharge rate, and higher one-year mortality. Moreover, low muscle mass was identified as a risk factor for one-year mortality among those with hip fracture. The aforementioned findings may help clinicians better manage those with hip fracture.

## Introduction

Age-related loss of muscle mass, called sarcopenia, a term proposed by Rosenberg et al. in 1989 [1], has been recognized as an independent condition by the International Classification of Disease, Tenth Revision [2]. Sarcopenia can be attributed to aging, undernutrition, disuse, and inflammation, resulting in functional decline, loss of independence, and early mortality among older individuals [3]. Sarcopenia has been identified as a risk factor for falls among older individuals, while patients with sarcopenia suffer from increased incidences of fractures [4, 5]. Indeed, Hida et al. reported that sarcopenia was a risk factor for hip fracture [6], which also affect activities of daily living and mortality among older individuals. Another study found that half of the patients with hip fracture ultimately develop permanent disability and mobility and are at high risk of institutionalization [7]. Mortality rates among those suffering from hip fracture had been reported to exceed 10% [8, 9], with increases rates observed within the first year after injury [10, 11]. The correlation between muscle mass and bone mass has been well known, with combined cases of sarcopenia and osteoporosis being common [12]. However, little is known regarding the impact of muscle mass on hip fracture treatment [13–15]. Therefore, the current study aimed to investigate the characteristics of patients with low muscle mass and determine the impact of muscle mass on hip fracture treatment outcomes.

## Methods

**Fig. 1:**
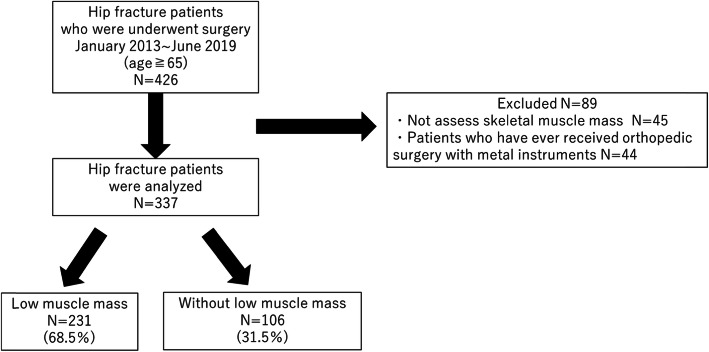
Flowchart showing progress through the study of the elderly patients with hip fracture

This case–control study involved patients with hip fracture aged ≥65 years who underwent orthopedic surgery from January 2013 to June 2019 in public hospital. Upon admission, all patients were measured for skeletal muscle mass index (SMI) and bone mineral density using dual-energy X-ray absorptiometry (Lunar iDXA; GE Healthcare, Tokyo, Japan). To avoid measurement errors in muscle mass and bone mineral density, 44 patients who had undergone orthopedic surgery with metal implants were excluded. Moreover, 45 patients who did not have skeletal muscle mass data on admission were excluded, considering the possibility of muscle mass loss due to lying in bed (Fig. 1). Low muscle mass was defined as the loss of appendicular skeletal muscle mass (ASM) (i.e., skeletal muscle mass in the arms and legs), with the SMI being calculated as ASM/height^2^ (kg/m^2^) according to the consensus of the Asian Working Group for Sarcopenia criteria (male, < 7.00 kg/m^2^; female, < 5.40 kg/m^2^) [16]. Walking speed could not be measured due to the presence of fractures, while grip strength could not be measured due to the inability of maintaining a standing or sitting position, an intravenous catheter in the dominant hand, and cognitive impairment in half of the patients. Osteoporosis was defined as a T score of ≤ − 2.5 standard deviations in the femoral neck without fracture. The presence of a dementia diagnosis was assessed using electronic medical record information and interviews. Hip fracture was classified as a femoral neck or trochanteric fractures. Characteristics and treatment outcomes were compared between both patients with and without low muscle mass. Nutritional status was assessed using the geriatric nutritional risk index (GNRI) [17], which was calculated using the following formula: 14.89 × serum albumin (g/dL) + 41.7 × [body weight (kg)/ideal body weight (kg)]. The ideal body weight was defined as that which resulted in a body mass index (BMI) of 22. GNRI was classified into the following four grades of nutrition-related risk: < 82, major risk; 82 to < 92, moderate risk; 92 to ≤98, low risk; and > 98, no risk. Treatment outcomes were assessed using stays at acute care institutions, hospital mortality, the Barthel index [18] at discharge, home discharge rate, and one-year mortality. Statistical analyses consisted of Student’s t-test for continuous variables, the Mann–Whitney U test for non-continuous variables, and the Pearson Chi-Square test for categorical variables. A multivariate logistic regression model was used to calculate adjusted odds ratios (ORs) with 95% confidence intervals (CIs) for factors related to low muscle mass. Kaplan–Meier survival curves on one-year mortality of hip fracture patients for those with and without low muscle mass were prepared, and log-rank tests were performed. Furthermore, Cox proportional hazards analysis adjusted for age and sex was performed to calculate adjusted hazard ratios (HRs) with 95% CIs for one-year mortality. All statistical analyses were performed using IBM SPSS v.23.0 for Windows (IBM Institute, Inc., Cary, NC, USA), with *P* < 0.05 indicating statistical significance. This study was approved by National Center for Geriatrics and Gerontology review board and all experiments were performed in accordance with the ethical standards laid down in the amened Declaration of Helsinki. This study was conducted with the ethics committee of National Center for Geriatrics and Gerontology (approval number: No. 1124). Informed consent was obtained from all individual participants included in the study.

## Results

**Fig. 2:**
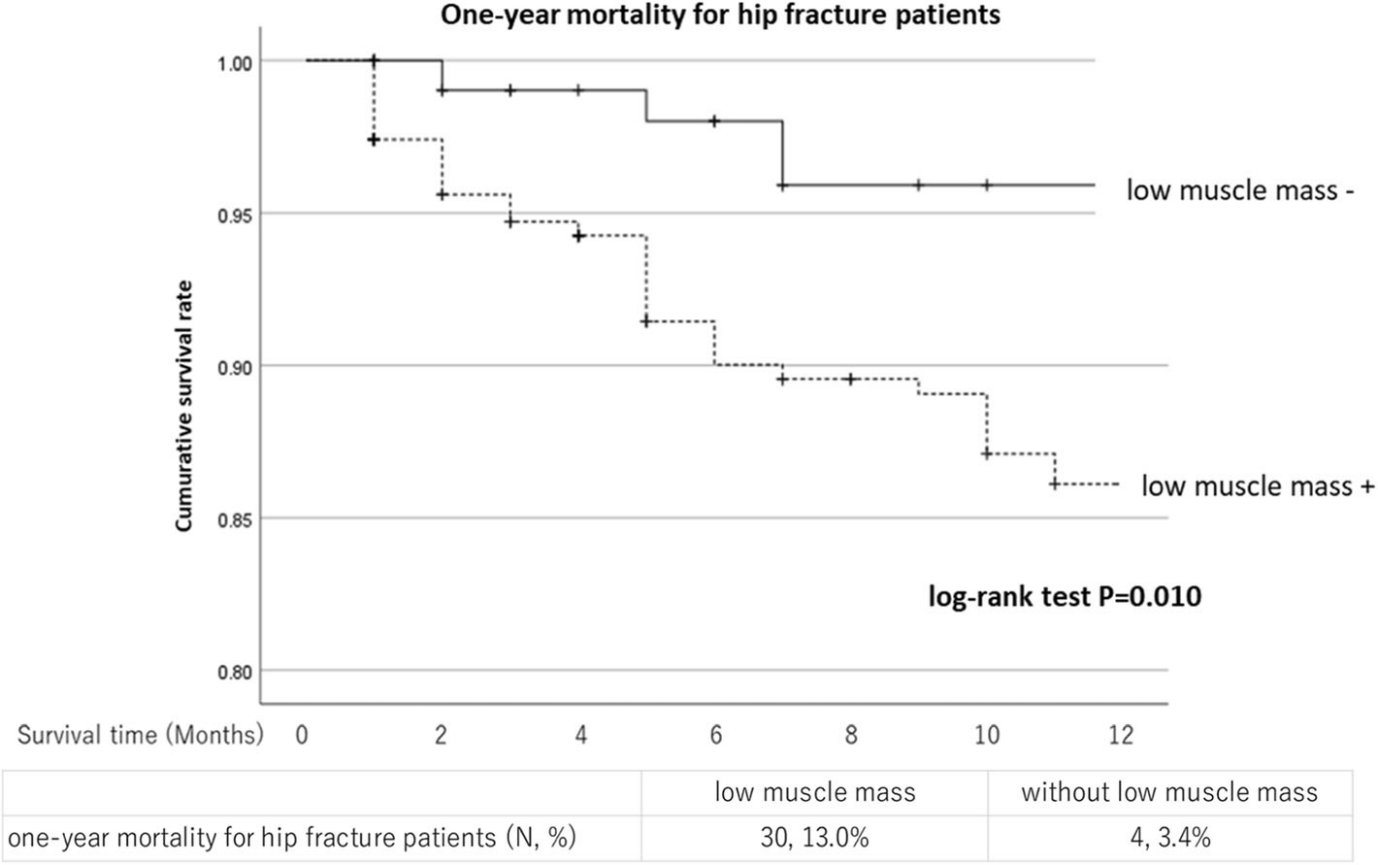
Kaplan-Meier survival curves of one-year mortality of hip fracture patients for those with and without low muscle mass

The total number of patients with hip fracture was 337 (67 males, 270 females) with a mean age of 84.1 ± 7.1 (65–102) years. None of the patients had high-energy trauma, and all the cases were caused due to minor traumas such as falling while walking or falling from a chair. Patients with neck fractures underwent total hip arthroplasty (3 cases), hemi hip arthroplasty (90 cases), and screw fixation (52 cases). All patients with trochanteric fractures underwent intramedullary fixation (192 cases). The mean follow-up period was 17.1 ± 14.5 (1–60) months. The prevalence of low muscle mass among patients with hip fracture was 231 cases (68.5%). With regard to patient characteristics, those with low muscle mass were predominantly male, had more femoral neck fractures, and had lower BMI, Barthel index, and GNRI. (*P* < 0.0001, *P* = 0.006, *P* < 0.0001, *P* = 0.019, *P* < 0.0001, respectively) (Table 1). Multivariate logistic regression analysis was performed to determine factors associated with low muscle mass, excluding SMI as an explanatory variable. Accordingly, male sex, low BMI, low GNRI, femoral neck fractures were associated with low muscle mass (OR 9.166, 95% CI 3.193–26.315, *P* < 0.0001; OR 0.719, 95% CI 0.622–0.830, *P* < 0.0001; OR 0.952, 95% CI 0.910–0.996, *P* = 0.033; OR 2.112, 95% CI 1.113–4.006, *P* = 0.022, respectively) (Table 2). Patients with low muscle mass had a lower Barthel index (*P* < 0.0001) and hospital discharge rate (*P* = 0.035) than those without low muscle mass (Table 3). Figure 2 shows the Kaplan–Meier survival curves for one-year mortality of hip fracture patients for those with and without low muscle mass, with the former having a higher one-year mortality than the latter (*P* = 0.011).

**Table 1:** Comparison of the patient characteristics for hip fracture with and without low muscle mass

Variables	Low muscle mass *N* = 231			Without low muscle mass *N* = 106			*P*-value		
Sex	male *N* = 61	female *N* = 170	total	male *N* = 6	female *N* = 100	total	male	female	total < 0.0001
Age (years)	82.4 ± 7.0	84.3 ± 7.1	83.8 ± 7.1	80.5 ± 8.1	85.0 ± 6.9	84.7 ± 7.0	0.307	0.616	0.262
BMI (kg/m^2^)	19.6 ± 3.1	19.1 ± 2.8	19.2 ± 2.9	23.5 ± 3.8	22.6 ± 3.0	22.7 ± 3.0	0.004	0.0001	< 0.0001
Cognitive impairment (N, %)	28, 45.9%	85, 50.0%	113, 48.9%	1, 16.7%	49, 46.2%	50, 47.2%	0.148	0.737	0.635
Home residence (N, %)	46, 75.4%	112, 65.9%	158, 68.4%	5, 83.3%	76, 76.0%	81, 76.4%	0.664	0.081	0.132
Barthel index (before injury)	70.5 ± 32.7	67.9 ± 30.7	68.5 ± 31.2	81.7 ± 27.3	76.7 ± 25.2	77.2 ± 25.3	0.426	0.02	0.019
GNRI	90.2 ± 11.2	89.8 ± 9.5	89.9 ± 9.9	100.9 ± 11.4	99.0 ± 8.7	99.2 ± 8.8	0.03	0.0001	< 0.0001
Osteoporosis (N, %)	37, 60.7%	145, 85.3%	182, 78.8%	2, 33.3%	73, 72.3%	75, 70.8%	0.195	0.013	0.108
Femoral neck fracture (N, %)	24, 39.3%	87, 51.2%	111, 48.1%	5, 83.3%	29, 29.0%	34, 32.1%	0.038	0.001	0.006
SMI (kg/m^2^)	5.40 ± 0.70	4.68 ± 0.49	4.87 ± 0.64	7.08 ± 1.16	6.08 ± 0.57	6.14 ± 0.65	< 0.0001	< 0.0001	< 0.0001

Values are presented as mean ± standard deviation

*BMI* Body mass index, *GNRI* Geriatric nutritional risk index, *SMI* Skeletal mass index

**Table 2:** The logistic regression analysis for related factors of low muscle mass

Variables	Odd ratio	95% CI	*P*-value
Age	0.984	0.939–1.031	0.498
Male sex	9.166	3.193–26.315	< 0.0001
BMI	0.719	0.622–0.830	< 0.0001
Barthel index (before injury)	0.991	0.980–1.002	0.108
GNRI	0.952	0.910–0.996	0.033
Femoral neck fracture	2.112	1.113–4.006	0.022

The dependent variable was the presence of low muscle mass

The presence of low muscle mass was attributed a value of 1, the absence of low muscle mass was attributed a value of 0

The male sex was attributed a value of 1, the female sex was attributed a value of 0

Femoral neck fracture was attributed a value of 1, trochanteric fracture was attributed a value of 0

*BMI* Body mass index, *GNRI* Geriatric nutritional risk index, *OR* Odds ratio, *CI* Confidence interval

**Table 3:** Comparison of the treatment outcomes for hip fracture with and without low muscle mass

	Low muscle mass (*N* = 231)	Without low muscle mass (*N* = 106)	*P*-value
Stays at acute care institutions (days)	28.8 ± 18.6	28.3 ± 14.3	0.811
Hospital mortality (N, %)	4, 1.7%	0, 0%	0.173
Barthel index (at discharge)	48.9 ± 32.4	61.7 ± 31.0	< 0.001
Home discharge (N, %)	111, 48.1%	64,60.4%	0.035

Values are presented as mean ± standard deviation

Furthermore, Cox proportional hazards regression analysis adjusted for age and sex revealed that low muscle mass was a risk factor for one-year mortality (HR 3.182, 95% CI 1.097–9.226, *P* = 0.033) (Table 4).

**Table 4:** Cox proportional hazards analysis adjusted for age and sex for one-year mortality of low muscle mass

Variables	HR	95% CI	*P*-value
Age	1.048	0.995–1.103	0.076
Male sex	2.018	0.978–4.166	0.057
Low muscle mass	3.182	1.097–9.226	0.033

Death within one-year was attributed a value of 1, survival after one-year was attributed a value of 0

The male sex was attributed a value of 1, the female sex was attributed a value of 0

The presence of low muscle mass was attributed a value of 1, the absence of low muscle mass was attributed a value of 0

*HR* Hazards ratio, *CI* Confidence interval

## Discussion

Rosenberg proposed sarcopenia as age-related muscle mass loss [1]. Given that sarcopenia promotes functional decline, loss of independence, and earlier mortality among older individuals [3], screening for patients with sarcopenia is imperative. Our study assessed only muscle mass. We defined low muscle mass, according to the Asian Working Group for Sarcopenia criteria (male, SMI < 7.00 kg/m^2^; female, SMI < 5.40 kg/m^2^). We could not measure walking speed and grip strength due to the presence of fractures, the inability of maintaining a standing or sitting position, an intravenous catheter in the dominant hand, and cognitive impairment in half of the patients.

The present study found that 68.5% of the included patients with hip fracture had low muscle mass (91.0% in males and 63.1% in females) and that male sex, underweight, undernutrition, and femoral neck fractures were associated with low muscle mass. Several studies have reported a higher prevalence of sarcopenia in men with hip fracture [6, 19]. Considering that males have more muscle mass than females, they may be more susceptible to the effects of age-related loss of muscle mass.

Underweight and undernutrition have been known risk factors for sarcopenia [3, 20]. Furthermore, while the type of hip fracture has been associated with age, sex, and bone mineral density [21–23], no study has yet investigated the relationship between the type of hip fracture and sarcopenia. Moreover, sarcopenia can be a negative prognostic predictor for patients with cancer [24]. However, little is known regarding the impact of sarcopenia on hip fracture management. Previous studies have reported that sarcopenia promotes poor functional outcomes after surgery and increases the risk of five-year mortality in patients with hip fracture [13–15].

Indeed, the present study found that patients with low muscle mass had a lower Barthel index, lower hospital discharge rate, and higher one-year mortality rate, which remains consistent with those presented in previous studies. These findings can potentially help clinicians make better treatment decisions and provide more information regarding surgical management to the patients and their families.

Our study found that the type of hip fracture was related to muscle mass. Notably, one study showed that patients with trochanteric fractures had lower bone mineral densities than those with femoral neck fractures [23], while another found a correlation between muscle mass and bone mass [12]. Therefore, we expected higher rates of trochanteric fractures among the low muscle mass group. However, the low muscle mass group had higher rates of femoral neck fractures than trochanteric fractures. Only a few studies have investigated the relationship between body composition and type of hip fracture. Among them, Di Monaco et al. reported that patients with femoral neck fractures had higher body fat mass than those with trochanteric fractures [25]. The difference between femoral neck and trochanteric fractures lies within muscle attachment considering that the magnitude of the reaction force applied to the bone caused by muscle contractions may affect the type of fracture. Nonetheless, further studies are needed to determine the relationship between muscle mass and type of hip fracture.

No consensus has been established regarding the treatment for low muscle mass. However, studies have shown that the combination of exercise training and nutritional supplementation can effectively improve muscle mass [26]. Exercise training, even at low intensity, has been shown to reduce mortality among older individuals [27]. As such, patients with hip fracture should be considered nutritional intervention and continue to exercise as much as possible after discharge. While no therapeutic agents are currently available for the treatment of low muscle mass, drugs utilized for the treatment of osteoporosis, such as alendronate and alfacalcidol, have been reported to have positive effects on muscle volume [28, 29]. However, given that these studies were conducted in the general population, it remains unclear whether similar results would be obtained in patients with hip fracture. Furthermore, gaining muscle mass does not prevent aging-related loss of muscle strength [30]. Bimagrumab (BYM338; Novartis), a fully human monoclonal antibody that prevents ligand binding and promotes differentiation of human myoblasts [31], has shown promising results in the treatment of sarcopenia. Studies have shown that although bimagrumab promoted greater muscle mass compared to placebo, no improvements in physical function were noted [32]. Further studies are therefore needed to develop an effective drug for the treatment of sarcopenia.

The presented study has several limitations worth noting. First, walking speed and grip strength could not be measured given the difficultly of evaluating physical function in patients during the acute phase of fractures. Although the diagnosis of sarcopenia requires assessing walking speed and grip strength, the current diagnostic criteria are controversial given that they exclude patients with locomotor disease (e.g., osteoarthritis, osteoporosis, and lumbar spinal stenosis). Sakai et al. reported that sarcopenia among older patients with locomotor disease (osteoarthritis, spondylosis, and osteoporosis) should be evaluated using muscle mass alone without physical performance [33]. Considering that most cases of fractures in older individuals are caused by falls and that most patients with hip fracture have osteoporosis, it may be reasonable to conclude that patients with hip fracture have impaired physical function. However, our patients had just low muscle mass in current diagnostic criteria of sarcopenia. Thus, we concluded low muscle mass affect hip fracture treatment outcomes in older individuals.

Second, the current study did not assess comorbidities (e.g., cancer, cardiac diseases, endocrine diseases, and neurological disease). Given that some patients had dementia or no relatives, a common occurrence in actual clinical practice, accurate medical histories could not be obtained. These comorbidities may have affected the treatment outcomes. However, given that these comorbidities also affect muscle mass loss (secondary sarcopenia), the diagnosis of sarcopenia may help assess the severity of these comorbidities. Third, we could not analysis separately for male and female, due to the small number of male hip fracture patients. Further research is needed to accumulate the number of cases.

## Conclusions

In summary, the current study identified male sex, underweight, undernutrition, and femoral neck fractures as factors associated with low muscle mass in patients with hip fracture. Moreover, among patients with hip fracture, those with low muscle mass had a lower Barthel index, lower hospital discharge rate, and higher one-year mortality. Furthermore, low muscle mass was identified as a risk factor for the one-year mortality among those with hip fracture. The aforementioned findings may help clinicians in the management of patients with hip fracture.

## Data Availability

The datasets analyzed during the current study available from the corresponding author on reasonable request.
